# Seasonally varying effects of environmental factors on phytoplankton abundance in the regulated rivers

**DOI:** 10.1038/s41598-019-45621-1

**Published:** 2019-06-25

**Authors:** Jun Song Kim, Il Won Seo, Donghae Baek

**Affiliations:** 1 Department of Earth Sciences, University of Minnesota, Minneapolis, MN 55455 USA; 2 Department of Civil and Environmental Engineering, Seoul National University, Seoul, 08826 South Korea; 3 Institute of Engineering Research, Seoul National University, Seoul, 08826 South Korea

**Keywords:** Hydrology, Ecological modelling

## Abstract

This study investigates a seasonally varying response of phytoplankton biomass to environmental factors in rivers. Artificial neural network (ANN) models incorporated with a clustering technique, the clustered ANN models, were employed to analyze the relationship between chlorophyll a (*Chl-a*) and the explanatory variables in the regulated Nakdong River, South Korea. The results show that weir discharge (*Q*) and total phosphorus (*TP*) were the most influential factors on temporal dynamics of *Chl-a*. The relative importance of both variables increased up to higher than 30% for low water temperature seasons with dominance of diatoms. While, during summer when cyanobacteria predominated, the significance of *Q* increased up to 45%, while that of *TP* declined to about 10%. These tendencies highlight that the effects of the river environmental factors on phytoplankton abundance was temporally inhomogeneous. In harmful algal bloom mitigation scenarios, the clustered ANN models reveals that the optimal weir discharge was 400 m^3^/s which was 67% of the value derived from the non-clustered ANN models. At the immediate downstream of confluence of the Kumho River, the optimal weir discharge should increase up to about 1.5 times because of the increase in the tributary pollutant loads attributed to electrical conductivity (*EC*).

## Introduction

Riverine ecosystems are significantly impacted by consequences of human activities such as effluents introduced from wastewater treatment plants and flow regulation associated with hydraulic structures even if rivers serve as essential sources of drinking water and provide habitats for freshwater fishes and invertebrates^1,2^. Phytoplankton have commonly been used as ecological indicators to assess these human effects on freshwater environments because phytoplankton blooms are usually results of excessive nutrient loading and extended water residence time induced by the artificial flow control^3,4^.

During the summer season, surface water quality is prone to be contaminated by cyanobacterial blooms that degrade water clarity, and even produce a variety of taste-and-odor compounds and toxins^5^. From early winter to late spring, high accumulation of diatoms adversely affects water intake activities by causing filter clogging^6^. For monitoring phytoplankton abundance, chlorophyll a (*Chl-a*) has widely been accepted as a measure of phytoplankton population in rivers and lakes^7^. Therefore, it is indispensable to understand a relationship between *Chl-a* and river environmental factors in order to predict its seasonal fluctuation and prepare countermeasures against phytoplankton blooms.

The *Chl-a* dynamics is conventionally simulated using numerical models based on an advection-dispersion-reaction equation. These kinds of physics-based models usually require not only extensive data related to boundary conditions, bathymetry and parameter estimation but also specialized knowledge of physical processes accounting for fate and transport of various water quality constituents^8^. As an alternative to the numerical approaches, the numerous studies have increasingly adopted data-driven techniques leveraged on artificial neural networks (ANNs) for modeling water quality^9–11^. Olden (2000) reported that *Chl-a* abundance was directly associated with nutrient and zooplankton variables through the ANN application to the prediction of phytoplankton succession^12^. Jeong *et al*. (2001, 2006) and Kim *et al*. (2014) evaluated the variable contribution of the ANN models to the predicted *Chl-a*, and their results indicated that *Chl-a* was most sensitive to pH and chemical oxygen demand (COD) rather than growth limiting factors generally known as water temperature, phosphorus and nitrogen^13–15^. Wu *et al*.^16^ also documented that total phosphorus and dissolved inorganic nitrogen considerably influenced the daily dynamics of *Chl-a* by performing sensitivity analysis with explanatory variables of the ANN models^17^.

The previous studies hypothesized that the relationship between *Chl-a* and the explanatory variables is seasonally homogeneous. In riverine and lacustrine systems, high water temperature favors cyanobacterial growth, whereas diatoms usually proliferate at low water temperature^14,18^. Thus, the transition of dominant phytoplankton species occurs with change in water temperature. Here, each of these phytoplankton groups exhibits its own growth characteristic as different species have different values of growth rate, half-saturation constant of nutrients such as phosphorus, nitrogen and silica, and optimal water temperature and light intensity^19–22^. Hence, an invariant stimulus can result in heterogeneous responses of *Chl-a* due to the inherent growth features inherent of phytoplankton species predominated in the water body, which changes with water temperature.

The conventional ANN models usually adopted water quality constituents that are byproducts of phytoplankton bloom like dissolved oxygen (DO), COD, turbidity and pH, as estimators of *Chl-a*^23^. In water bodies, phytoplankton produce and consume DO through photosynthesis and respiration, respectively. Simultaneously, phytoplankton detritus increases COD and herein resulted in DO depletion through bacterial decomposition^24^. In addition, the rapid accumulation in phytoplankton biomass degrades water transparency with increasing turbidity and cause increase in pH by consuming hydrogen during the photosynthesis^25,26^. However, the ANN modeling with the above water quality variables not physically affecting the growth dynamics of phytoplankton could be prone to mask or distort the cause and effect relationship between *Chl-a* and the growth limiting factors such as water temperature, residence time and nutrient concentrations.

This study is aimed at understanding seasonally varying response of *Chl-a* to the river environmental factors that directly contribute to phytoplankton growth mechanism in riverine systems. In this work, ANN models incorporated with a cluster technique (clustered ANN models) were used to consider the effects of seasonal transition of phytoplankton communities on relationships between *Chl-a* and input variables by partitioning field data according to different ranges of water temperature. The model performance was evaluated by comparison with that of the conventional ANN model without clustering. Using the clustered ANN models, this study estimated the relative importance of the environmental variables on *Chl-a* prediction and furthermore performed the scenario-based simulations to propose an optimal flow condition for suppressing the phytoplankton bloom in regulated rivers, where flow discharge is artificially controlled by hydraulic structures.

## Materials and Methods

### Study area

The Nakdong River is one of the major rivers in South Korea, which is about 525 km long and includes large tributaries including the Kumho River. This large river is served as important water supply sources for about 10 million residents in the south eastern area, passing through the major cities of Busan and Daegu. The drainage area is 23,817 km^2^ with an average channel width and water depth of about 250 m and 7.4 m, respectively. The 5-year average precipitation of the Nakdong River watershed was 958 mm during 2013–2017, and 18.4% and 1.9% of the total watershed area are used as agricultural and industrial complex areas, respectively. The non-point source with rainfall-runoff events contributes to about 60% of total phosphorus loading in the study area^27^.

**Figure 1:**
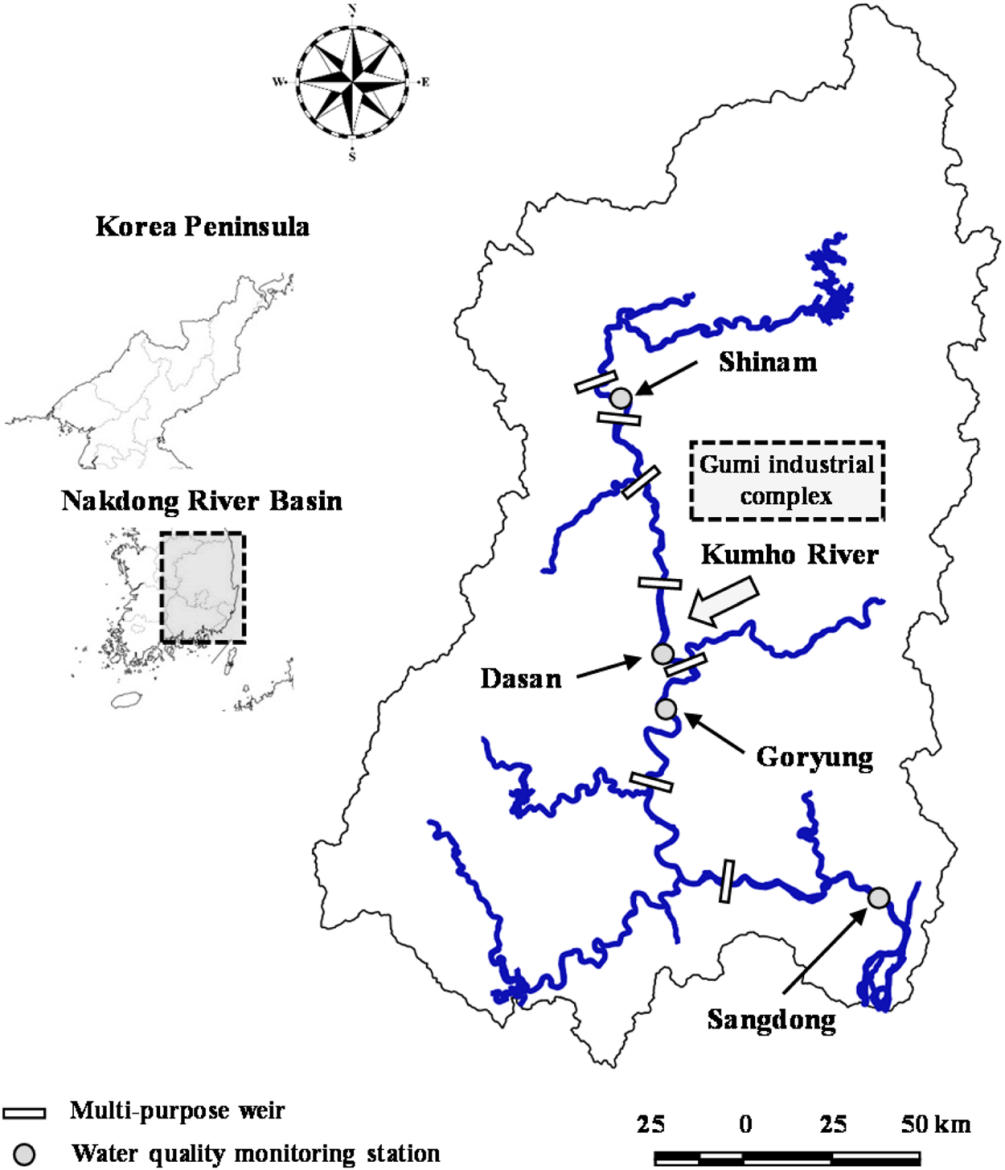
Location of target water quality monitoring stations in the Nakdong River.

Corresponding to the latest climate change, multi-purpose weirs were constructed across the Nakdong River in 2012 to achieve the flood protection and drought combat, as shown in Fig. 1. After the weir construction, this regulated river has suffered the aberrant proliferation of phytoplankton with the change in seasonal patterns of river flow^28^. In the regulated Nakdong River, toxic cyanobacteria such as *Microcystis*, *Aphanizomenon* and *Anabaena* have been abundant, and herein resulted in the significant level of cyanobacterial cell counts around 10,000–20,000 cells/ml during summer owing to the water temperature higher than 25 °C and the extended water residence time arisen from the artificial flow control by the weirs^29^. From winter to early spring, diatoms with the prevalence of *Stephanodiscus* have usually predominated in the study area^30^.

### Field data

This study selected water temperature (*WT*), total phosphorus (*TP*) and total nitrogen (*TN*) as predictors of *Chl-a* in order to only take into account water quality factors physically associated with the phytoplankton growth dynamics. The flow velocity representing water residence time can moreover be deemed to the significant factor for predicting the phytoplankton abundance. The extended water residence time attributed to flow velocity facilitates the phytoplankton accumulation as well as stabilizes the water column to result in the thermal stratification, which favors cyanobacterial bloom during summer, in the regulated rivers^31,32^. In the Nakdong River, the weirs has not only fixed water surface elevation to the design level but also controlled flow rate using movable weir gates. Herein, flow velocity of the river can be estimated by the discharge from the weirs since the water surface elevation and channel width remain constant. For this reason, this study additionally considered weir discharge (*Q*) as the hydraulic predictor of *Chl-a*.

In the midstream of the Nakdong River, the large urban-industrial complexes are located along the tributaries, where wastewater effluents containing excessive loadings of phosphorus and nitrogen are continuously spilled into the Nakdong River. Due to this tributary-driven contamination, the water quality after the confluence of the tributaries has severely deteriorated^33^. Especially, phytoplankton blooms have frequently propagated from the merging point of the Kumho River which is the largest tributary located in the middle reach of the Nakdong River^34^. The 5-year average electrical conductivity (*EC*) and *Chl-a* of the Kumho River are 710 μS/cm (678–780 μS/cm) and 48.8 mg/m^3^ (35.6–61 mg/m^3^), respectively for 2013–2017. These levels are higher than twice the values observed at the monitoring stations, particularly Dasan station which is located before the confluence of this tributary, as shown in Table 1. Here, *EC* can be the appropriate indicator to explain the tributary effects on the seasonal variation of *Chl-a* in the study area because the tributary influx, which is the external source of *Chl-a*, usually exhibits high concentration of *EC* induced by the industrial facilities^35^. Therefore, *WT*, *EC*, *TN*, *TP* and *Q* were determined as the input variables of the ANN models to derive the relationship between the river environmental factors and *Chl-a* using the field-based monitoring data.

**Table 1:** Daily water quality and discharge data collected at monitoring stations in the Nakdong River for 5 years (2013–2017).

Sites	*WT* (°C)	*EC* (μS/cm)	*TN* (mg/L)	*TP* (mg/L)	*Q* (m^3^/s)	*Chl-a* (mg/m^3^)
Shinam (*n* = 1,474)	15.8 ± 8.9 (0.7–31.7)	216 ± 43 (103–378)	2.42 ± 0.65 (0.78–9.21)	0.021 ± 0.018 (0.002–0.136)	75 ± 109 (2–1,839)	18.3 ± 11.9 (0.4–92.9)
Dasan (*n* = 1,023)	18.9 ± 8.5 (2.4–33.5)	291 ± 72 (124–561)	2.63 ± 0.64 (0.88–4.26)	0.029 ± 0.015 (0.007–0.103)	117 ± 221 (2–3,153)	19.2 ± 13.2 (0.1–125.2)
Goryung (*n* = 1,031)	17.6 ± 8.6 (2.9–33.6)	416 ± 121 (144–802)	3.85 ± 0.83 (1.59–6.20)	0.035 ± 0.023 (0.006–0.142)	120 ± 208 (3–2,154)	26.7 ± 21.2 (1.2 ± 152.2)
Sangdong (*n* = 1,475)	16.9 ± 8.7 (1.7–33.0)	288 ± 78 (124–503)	2.47 ± 0.64 (0.76–6.07)	0.024 ± 0.016 (0.003–0.102)	266 ± 348 (14–3,734)	25.7 ± 16.9 (1.0 ± 109.4)

The ANN models were constructed at several water quality monitoring stations such as Shinam station, Dasan station, and Sangdong station located at upstream, midstream and downstream of the Nakdong River, respectively, as shown in Fig. 1. The 5-year (2013–2017) daily data for *WT*, *EC*, *TN*, *TP* and *Chl-a* were available at these monitoring stations, and the corresponded data for *Q* were retrieved from the streamflow gauge stations at the multi-purpose weirs located upstream of each monitoring station. Goryung station was additionally selected as the prediction site, which is located downstream of the confluence of the Kumho River, to analyze the contribution of the tributary effluents with the high concentration of *EC* introduced from the tributary to the phytoplankton biomass. The input and output data of the ANN models are presented in Table 1. From this table, one can notice the abrupt rise in average values of *EC*, *TN*, *TP* and *Chl-a* at Goryung station due to the tributary effect.

### Model description

The architecture of ANN models was composed of the input layer, the single hidden layers, and output layer. In these networks, the input variables in the input layer were multiplied by the weight factors to link the connections between the input layer and the hidden layer, and then the bias was added as:

1 $${H}_{j}=\sum _{i=1}^{N}{x}_{i}{W}_{ij}+{b}_{j}$$

where *i* indicates the node in the input layer; *x*_*i*_ is the input variable at *i*; *j* indicates the node in the hidden layer; *W*_*ij*_ is the weight factors at *j*; *b*_*j*_ is the bias at *j*; and *N* is the number of nodes in the input layer. The values of input variable such as *WT*, *EC*, *TN*, *TP*, and *Q* had different ranges so that they were scaled to the uniform ranges using the min-max normalization technique as following:

2 $${X}_{i}=\frac{{x}_{i}-\,\min ({x}_{i})}{\max ({x}_{i})-\,\min ({x}_{i})}$$

where *X*_*i*_ is the normalized value of the input variables. *W*_*ij*_ is the key parameter of the ANN models, and these values were optimized during the training phase by minimizing the error between prediction and target. In this process, the initial values of *W*_*ij*_ were selected by Xavier initialization to initiate the training of the ANN model*s*, in which *b*_*j*_ was initially set to be zero^36^. Using this technique, the weight factors were randomly selected from the uniform distribution with the interval of $\pm 1/\sqrt{N}$.

The values of *H*_*j*_ in the hidden layer were then transferred to the output layer passing through the activation function. With this treatment, the ANN models can generate the nonlinear relationship between the input and output values. This study adopted the tangent hyperbolic function as the activation function, which can be described as:

3 $$\delta ({H}_{j})=\,\tanh ({H}_{j})$$

The values processed with the activation function in the hidden layer were multiplied by the weight factors that are located between the hidden layer and the output layer. The output layer included the single node which indicates *Chl-a* predicted by the ANN models and can be obtained as:

4 $$\hat{C}=\sum _{j=1}^{M}\delta ({H}_{j}){W}_{jk}+{b}_{k}$$

where $\hat{C}$ is the predicted *Chl-a*; k denotes the node in the output layer; *W*_*jk*_ is the weight factor at *k*; *b*_*k*_ is the bias at *k*; and *M* is the number of the nodes in the hidden layer. The ANN models were optimized using the cost function which represents the sum of squared errors calculated as:

5 $${\rm{SSE}}=\frac{1}{2}\sum _{i=1}^{n}{({\hat{C}}_{i}-{C}_{i})}^{2}$$

where *n* is the number of data; and ${\hat{C}}_{i}$ and *C*_*i*_ are the *i*^th^ predicted and observed *Chl-a*, respectively. To minimize the cost function, all trainable parameters including the weight factors and the bias in the ANN models were updated at each epoch using the adaptive gradient descent algorithm^37^. In this process, the ANN models were trained using 70% of the total datasets to estimate the trainable parameters. The remaining 30% datasets were used for testing the trained ANN models. Using the optimized parameters, the contribution of the river environmental factors to the *Chl-a* prediction can be calculated as^38^:

6 $$R{I}_{i}=\sum _{i=1}^{N}\frac{|{W}_{ij}{W}_{jk}|}{\sum _{j=1}^{M}|{W}_{ij}{W}_{jk}|}\times 100 \% $$

where *RI*_*i*_ is the relative importance of the *i*^th^ input variable. This method determines the relative importance of each input variable in the ANN models by partitioning the neural network connection weights^39^.

### Data clustering

As aforementioned, the response of *Chl-a* to the explanatory variables changes according to the phytoplankton species dominant in rivers. For this reason, the ANN models need to be structured separately for capturing the temporally varying relationship between the river environmental factors and *Chl-a*. In the Nakdong River, the phytoplankton communities such as cyanobacteria and diatoms shifts seasonally so that their succession can be explained by the change of the water temperature^40^. However, it is not usually feasible to identify the thresholds of the water temperature for differentiating cyanobacteria and diatoms from the time-series data of *Chl-a*. Hence, the total datasets were partitioned into several clusters based on the water temperature by adopting K-means clustering in order to construct the multiple ANN models corresponding to the number of the clusters. This clustering technique is the algorithm to split the datasets into K clusters, in which the partitioned datasets belong to each cluster with the nearest mean. The partition of the datasets was processed by minimizing the sum of squares of distances between data and the corresponding cluster centroid as following^41^:

7 $$E={\sum _{l=1}^{K}\sum _{{x}_{i}\in {C}_{l}}\Vert {x}_{i}-{\mu }_{l}\Vert }^{2}$$

where *K* indicates the number of the clusters; *l* is the cluster; $\Vert {x}_{i}-{\bar{\mu }}_{l}\Vert$ is the Euclidean distance between *x*_*i*_ and *μ*_*l*_; *x*_*m*_ is the *i*^th^ observation data; and *μ*_*l*_ is the centroid of *l*^th^ cluster. Table 2 summarizes the statistics of the input and output data before and after clustering the datasets at Goryung station. In this table for K = 3, Cluster 31, Cluster 32 and Cluster 33 represent the datasets belonging to low, intermediate and high water temperature, respectively. The datasets collected at other monitoring stations were also clustered following the same manner as demonstrated in Table 2.

**Table 2:** Summary of clustered inputs (*WT*, *EC*, *TN*, *TP*, *Q*) and output (*Chl-a*) data collected at Goryung station.

Variables	Non-clustered ANN model	2-cluster ANN model		3-cluster ANN model		
	K = 1	K = 2		K = 3		
	Cluster 11 (*n* = 1,031)	Cluster 21 (*n* = 465)	Cluster 22 (*n* = 566)	Cluster 31 (*n* = 343)	Cluster 32 (*n* = 274)	Cluster 33 (*n* = 414)
*WT* (°C)	17.6 ± 8.6 (2.9–33.6)	9.1 ± 3.9 (2.9–16.5)	24.6 ± 3.9 (16.6–33.6)	7.2 ± 2.4 (2.9–12.2)	17.2 ± 2.7 (12.3–21.8)	26.5 ± 2.5 (21.9–33.6)
*EC* (μS/cm)	416 ± 121 (144–802)	483 ± 121 (176–802)	360 ± 89 (144–610)	503 ± 112 (270–802)	392 ± 104 (176–726)	359 ± 95 (144–610)
*TN* (mg/L)	3.85 ± 0.83 (1.59–6.20)	4.50 ± 0.61 (3.20–6.20)	3.32 ± 0.56 (1.59–4.68)	4.64 ± 0.55 (3.32–6.20)	3.76 ± 0.59 (2.72–5.37)	3.26 ± 0.59 (1.59–4.46)
*TP* (mg/L)	0.035 ± 0.023 (0.006–0.142)	0.024 ± 0.013 (0.006–0.090)	0.044 ± 0.026 (0.008–0.142)	0.023 ± 0.011 (0.007–0.068)	0.029 ± 0.017 (0.006–0.112)	0.048 ± 0.027 (0.008–0.142)
*Q* (m^3^/s)	120 ± 208 (3–2,154)	66 ± 55 (3–459)	164 ± 269 (3–2,154)	57 ± 49(3–459)	105 ± 120 (3–1,196)	181 ± 299 (3–2,154)
*Chl-a* (mg/m^3^)	26.7 ± 21.2 (1.2 ± 152.2)	29.1 ± 22.3 (5.3–138.2)	24.6 ± 19.9 (1.2–152.2)	28.6 ± 20.9 (5.3–136.6)	24.1 ± 20.0 (1.2–138.2)	26.7 ± 21.9 (1.5–152.2)

### Model performance criteria

To evaluate the performance of the trained ANN models by comparing predictions with observations, the statistical performance measures were used as follows:

8 $${{\rm{R}}}^{2}=1-\frac{\sum _{i=1}^{n}{({C}_{i}-{\hat{C}}_{i})}^{2}}{\sum _{i=1}^{n}{({C}_{i}-{\bar{C}}_{i})}^{2}}$$

9 $${\rm{RSR}}=\frac{\sqrt{\sum _{i=1}^{n}{({C}_{i}-{\hat{C}}_{i})}^{2}}}{\sqrt{\sum _{i=1}^{n}{({C}_{i}-{\bar{C}}_{i})}^{2}}}$$

10 $${\rm{APBIAS}}=\frac{\sum _{i=1}^{n}|{C}_{i}-{\hat{C}}_{i}|}{\sum _{i=1}^{n}{C}_{i}}\times 100 \% $$

*R*^2^ ranges from 0 to 1 and higher values indicate better model performance. RSR is ratio of RMSE and standard deviation of the observed data. APBIAS measures the average tendency of the prediction results to be more deviated than the observation data. The optimal value of RSR and PBIAS is 0, which indicates the perfect model prediction.

## Results and Discussion

### ANN models without clustering

**Figure 2:**
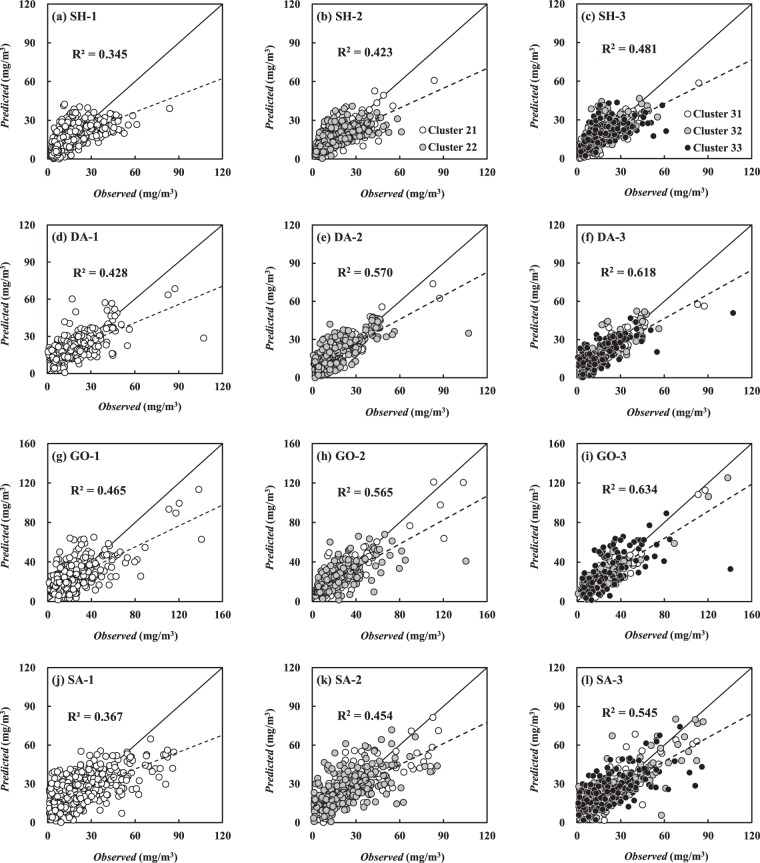
Testing results of *Chl-a* prediction with trained non-clustered, 2-cluster and 3-cluster ANN models (the solid line and dotted line indicate a 1:1 linear line and a fitting line to predictions, respectively).

Before implementing the ANN modeling incorporated with K-means clustering, the ANN models without clusters, the non-clustered ANN models (K = 1) were first constructed at Shinam station, Dasan station, Goryung station, and Sangdong station. This study employed k-fold cross validation to determine the optimal number of the hidden neuron for the ANN models, and the optimal number was 15 for all prediction sites. Table 3 summarizes the training and testing results at each monitoring station, in which SH-1, DA-1, GO-1, and SA-1 denote the non-clustered models for Shinam station, Dasan station, Goryung station and Sangdong station, respectively. According to this table, the trained ANN models showed *R*^*2*^ of 0.507–0.624 at the prediction sites. As a result of testing the trained ANN models, the prediction accuracy was *R*^*2*^ of 0.345, 0.428, 0.465, and 0.367 of at Shinam station, Dasan station, Goryung station and Sangdong station, respectively. Figure 2 is evident that the predicted values of *Chl-a* tended to be relatively underestimated compared to the observations at all prediction stations.

**Table 3:** Prediction accuracy of ANN models with different numbers of clusters.

Models	Training			Testing		
	R^2^	RSR	APBAIS (%)	R^2^	RSR	APBAIS (%)
SH-1	0.507	0.702	34.2	0.345	0.823	42.1
SH-2	0.639	0.601	28.7	0.423	0.771	38.7
SH-3	0.698	0.551	26.6	0.481	0.735	37.1
DA-1	0.551	0.673	32.2	0.428	0.775	37.2
DA-2	0.726	0.523	24.8	0.570	0.663	32.1
DA-3	0.754	0.497	23.8	0.618	0.623	30.6
GO-1	0.624	0.613	36.4	0.465	0.737	44.4
GO-2	0.772	0.478	28.2	0.565	0.661	35.9
GO-3	0.806	0.441	25.9	0.634	0.609	34.1
SA-1	0.550	0.671	32.7	0.367	0.805	42.1
SA-2	0.703	0.545	26.3	0.454	0.752	37.8
SA-3	0.698	0.549	26.2	0.545	0.682	34.5

### ANN models with clustering

The clustered ANN models were constructed by partitioning the input and output data into 2 and 3 cluster groups (K = 2 and 3) according to the ranges of the water temperature using K-means clustering. The optimal number of the hidden neuron was 15 for both ANN models with 2 and 3 clusters, referred to the 2-cluster ANN models and 3-cluster ANN models, respectively at the monitoring stations. Figure 2 indicates that the 2-cluster ANN models (SH-2, DA-2, GO-2 and SA-2) showed the enhanced prediction accuracy by comparison with that of the non-clustered ANN models. The underestimated predictions of *Chl-a*, caused by the non-clustered ANN models, was diminished with the 2-cluster ANN models. The prediction accuracy was even more improved when using the 3-cluster ANN models (SH-3, DA-3, GO-3 and SA-3) that distinctively minimized the deviations from the perfect linear line, as shown in Fig. 2 as the 3-cluster models partitioned the datasets into three different groups indicating the diatom season (Cluster 31), transition season (Cluster 32) and cyanobacteria season (Cluster 33). The training results of the 3-cluster ANN models showed *R*^*2*^ of 0.690–0.806, and the trained ANN models resulted in *R*^*2*^ of 0.485, 0.618, 0.634 and 0.545 at Shinam station, Dasan station, Goryung station and Sangdong station, respectively, as shown in Table 3. Moreover, RSR and APBIAS decreased by about 0.10–0.15 and 6–10%, respectively, compared to those resulted from the non-clustered ANN models.

**Figure 3:**
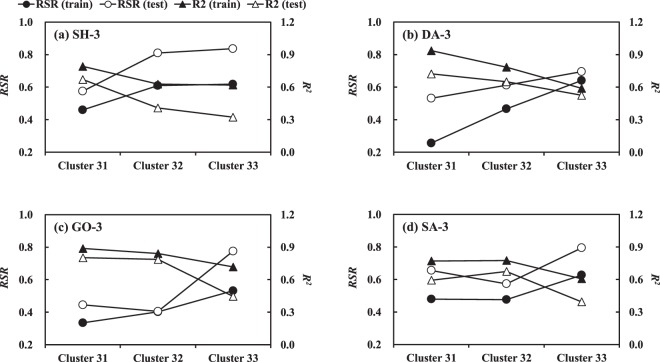
Prediction accuracy of 3-cluster ANN models in predicting *Chl-a* belonging to different clusters.

As a result of the 3-cluster ANN modeling, the prediction accuracy for *Chl-a* in Cluster 32 and Cluster 33 was generally lower than that in Cluster 31 at all monitoring stations, as shown in Fig. 3. Ha *et al*. (2003) and Kim *et al*. (2018a) reported that centric diatoms, *Stephanodiscus* usually occupy about 85% of total phytoplankton population in the Nakdong River from late fall to spring (about 5–15 °C)^18,30^. Thus, the ANN models adequately explained the seasonal behavior of *Chl-a* in the low water temperature below about 15 °C as the concentration of *Chl-a* in Cluster 31 was governed by the population of the single diatom assemblage. Whereas, in the warm seasons, the multiple communities of cyanobacteria such as *Microcystis*, *Anabaena* and *Aphanizomenon* constitute the biomass of total phytoplankton in the study area^42^. The complex dynamics of the cyanobacteria therefore hampered the accurate prediction for *Chl-a* belonging to Cluster 32 and Cluster 33.

### Relative importance of explanatory variables

**Figure 4:**
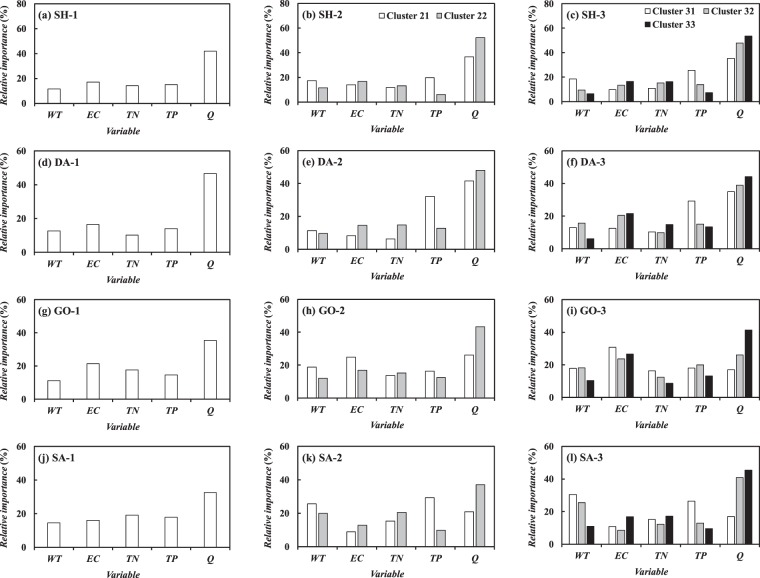
Relative importance of explanatory variables on predicted *Chl-a* belonging to different ranges of water temperature constituting each cluster in ANN models.

Figure 4 shows the relative importance of the explanatory variables on *Chl-a* prediction, which was calculated using Eq. (6), at the target stations using both non-clustered and clustered ANN models. According to Fig. 4(a,d,g,j), at all prediction sites, the non-clustered ANN models revealed that *Q* was the most dominant factor in predicting the seasonal variation of *Chl-a* as this hydraulic variable contributed 32.5–46.7% to the prediction. On the other hand, less than 20% was contributed to the prediction by each of other input variables such as *WT*, *EC*, *TN*, and *TP*. Since the multi-purpose weirs controlled the river discharge, *Q* accounted for the water residence time which affects the intensity and timing of the phytoplankton bloom, and thereby substantially influenced the seasonal dynamics of *Chl-a*.

Similar to the results of the non-clustered ANN modeling, with the 2-cluster ANN models, *Q* was the most influential input variable on the daily fluctuation of *Chl-a*. Here, the influence of Q was stronger in Cluster 22 than in Cluster 21 while its relative importance increased up to 52.7%, as depicted in Fig. 4(b,e,h,k). Among the nutrient variables, *TP* exhibited the high values of the relative importance up to 32.2% when predicting *Chl-a* belonging to Cluster 21. However, for the prediction of *Chl-a* in Cluster 22, the relative importance of *TP* drastically decreased to about 10% which was occasionally lower than that of *TN*. These results indicated that *TP* did not always act as the limiting nutrient for the phytoplankton growth despite it has generally been known that the phytoplankton growth in the freshwater systems was limited by TP concentration^43,44^. This seasonal variation in the relative importance of the river environmental factors was not captured with the conventional ANN approaches like the non-clustered ANN models that assume the temporal homogeneity in the input-output relationship.

The results with the 3-cluster ANN models revealed that the significance of *Q* was amplified with increase in water temperature as the reach-averaged relative importance of *Q* was 26.0, 38.4 and 46.2% for Cluster 31, Cluster 32 and Cluster 33, respectively. In the meanwhile, the significance of *TP* declined as water temperature increased as the relative importance of *TP* ranged 18.0–29.3%, 12.9–19.9% and 7.3–13.4% for Cluster 31, Cluster 32 and Cluster 33, respectively, as shown in Fig. 4(c,f,i,l). In the Nakdong River, *Chl-a* in Cluster 31 represents the diatom concentration while that in Cluster 33 usually constitutes the cyanobacterial biomass. Thus, from these results, one can notice that *TP* played a role as the limiting factor for the growth of not cyanobacteria but diatoms, while the seasonal variation of cyanobacteria was highly affected by the retention time rather than the nutrient concentration. The previous studies reported that the forming of cyanobacteria was strongly impacted by the artificial mixing stimulated by the increase in *Q* as the enhanced vertical mixing destructs the thermal stratification as well as disturbs the buoyancy mechanism to alleviate the intensity of the cyanobacterial bloom^4,45,46^. Hence, the results of the clustered ANN modeling elucidated that the relationship between *Chl-a* and the river environmental factors was temporally heterogeneous and dependent on the seasonal succession of the phytoplankton community.

The results also showed that the significance of the river environmental factors changed according to the location of the monitoring stations because the relationship between the target water quality and the explanatory variables can vary spatially due to the biogeochemical characteristics inherent to the specific locations^35^. As aforementioned, the water quality variables including *Chl-a* observed at Goryung station was strongly influenced by the effluents introduced from the Kumho River, which constitute the high concentration of *EC*. Figure 4(i) represents that the seasonal variation of *Chl-a* at this monitoring station was considerably explained by *EC* which contributed 23.6–30.8% to the prediction with the 3-cluster ANN models as the water quality of this hypereutrophic Kumho River acted as the external sources of *Chl-a*. Whereas, the relative importance of *EC* was almost less than 20% at other monitoring stations. The tributary inflow therefore needs to be regarded as the explanatory variable for the *Chl-a* prediction in the rivers including the confluence zone.

### Impact assessment of weir discharge on cyanobacterial bloom

To propose the countermeasures against toxic cyanobacterial bloom prevailing in summer, also known as harmful algal bloom (HAB) which severely impacts the ecosystem functioning in the Nakdong River, this study performed the scenario-based simulation using the 3- cluster ANN models. The mitigation approaches primarily focused on the effects of the weir discharge control on the reduction of HAB as *Q* was the dominant factor over other input variables in the prediction of *Chl-a* belonging to high water temperature (Cluster 33) at all monitoring stations. For the scenario generation, *Q* increased from 20 to 600 m^3^/s with the increment of 20 m^3^/s, and the remaining input variables were fixed to the average values of the datasets corresponding to Cluster 33 of each monitoring station.

**Figure 5:**
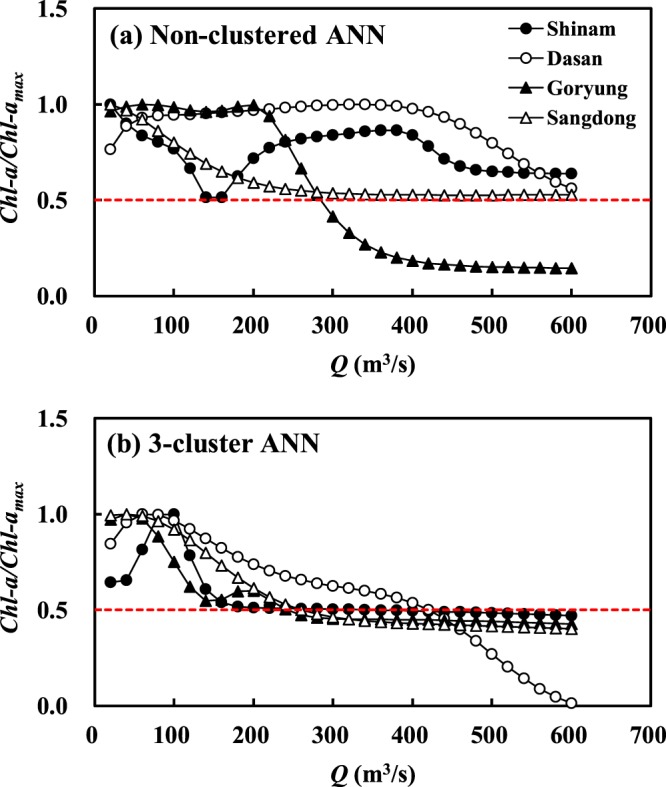
Response of *Chl-a* to increase in *Q*, estimated by non-clustered ANN models and 3-cluster ANN models.

As a result of the scenario-based simulation, with the non-clustered ANN models, the concentrations of *Chl-a* diminished to 50% of the maximum values at all monitoring stations when *Q* increased up to 600 m^3^/s, and no distinct change in *Chl-a* was simulated in the further increase in *Q*, as shown in Fig. 5(a). On the other hand, the 3-cluster ANN models demonstrated that the same level of the *Chl-a* reduction was obtained with *Q* of 400 m^3^/s which was only 67% of the amount estimated by the non-clustered ANN models, as illustrated in Fig. 5(b). Consequently, the non-clustered ANN tended to overestimate the optimal level of *Q* for the HAB control, which could mislead the flow management in the Nakdong River.

The seasonal fluctuation of *Chl-a* at the specific site like Goryung station was very sensitive to the change in *EC*, as demonstrated in Fig. 4(i). The additional scenario-based simulation was thus conducted to investigate the effect of *EC* on determining the optimal value of *Q* for the HAB reduction. The scenarios were prepared as follows; *Q* was set to 250–600 m^3^/s with the increment of 50 m^3^/s, and *EC* ranged 140–500 μS/cm with the increment of 20 μS/cm. Other explanatory variables were constant to the average values of the datasets belonging to Cluster 33 at all target sites, as shown in Table 3.

**Figure 6:**
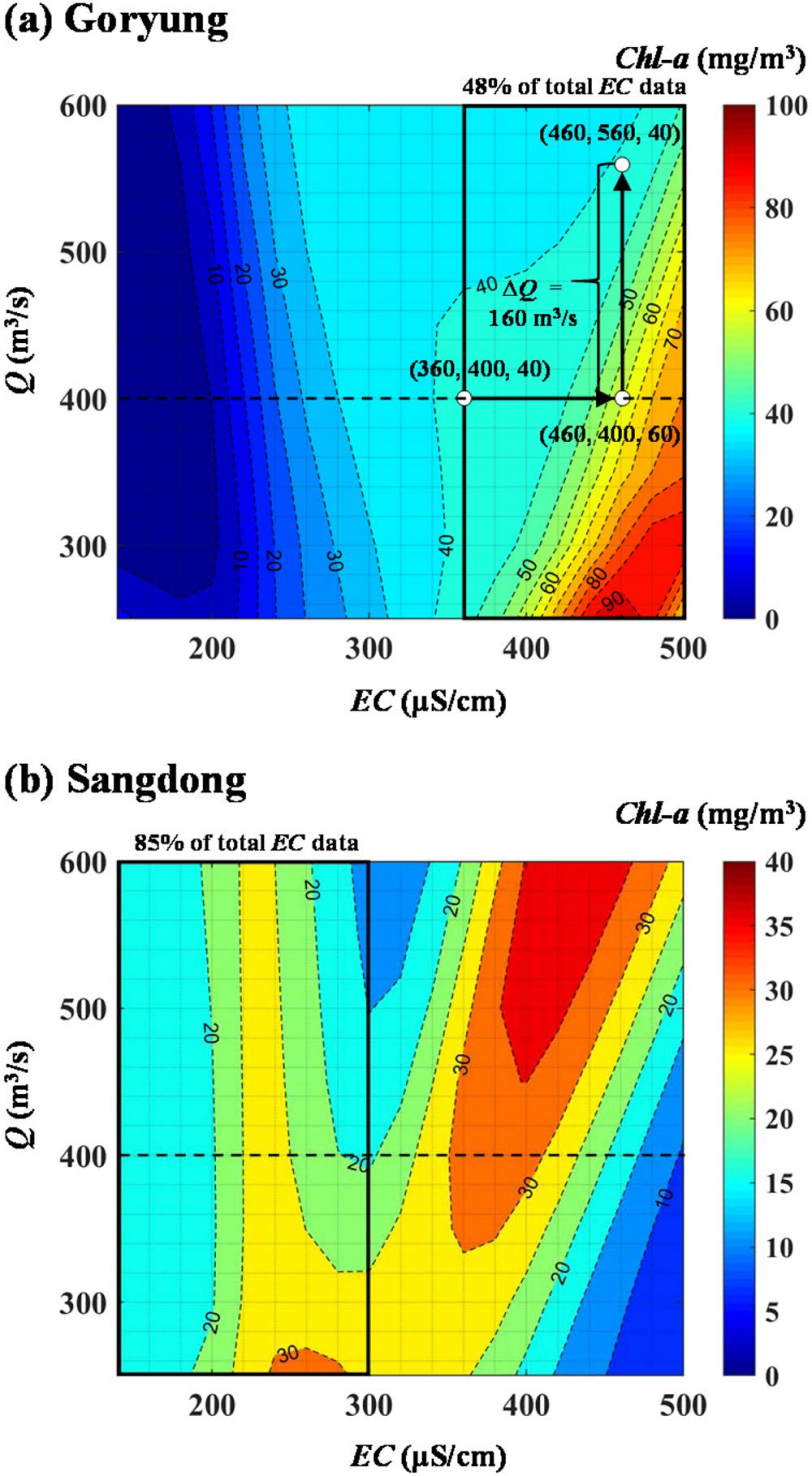
Response of *Chl-a* to increase in *EC* with different conditions of *Q* at Goryung station and Sangdong station, estimated by 3-cluster ANN models (a dotted line indicates the optimal level of *Q* derived from Figure 5).

The simulation results showed that, in the case of Goryung station, the additional amount of *Q* was required to control *Chl-a* with the increase in *EC*. Figure 6(a) indicates the abrupt rise in *Chl-a* in the region of *EC* ranging 360–600 μS/cm, which corresponds to 48% of the total *EC* data at the target site. For example, *Chl-a* of 40 mg/m^3^ was simulated when *EC* and *Q* were set to 360 μS/cm which is the average value of Cluster 33 and 400 m^3^/s derived from Fig. 5(b), respectively. However, if *EC* increased to 460 μS/cm, the corresponded amount of *Q* was 560 m^3^/s to retain the same value for *Chl-a* (40 mg/m^3^) as obtained in the former case. In contrast, in the case of Sangdong station with *Q* of 400 m^3^/s, *Chl-a* did not change significantly and remained relatively constant to around 15–25 m^3^/s in the range of *EC* less than 300 μS/cm, which corresponds to 85% of the total *EC* data at this monitoring station, as shown in Fig. 6(b). Hence, in the river reaches adjacent to the confluence of the contaminated tributaries, the water quality variable accounting for the tributary effluents should be considered as the crucial factor in predicting the phytoplankton abundance and furthermore assessing the optimal level of *Q* for the HAB mitigation during the summer season.

## Conclusions

This study applied the clustering technique to the ANN models for predicting the seasonal variation of *Chl-a* by capturing the relationship between the river environmental factors and *Chl-a* at the water quality monitoring stations located in the Nakdong River, of which the discharge was regulated by a number of the weirs. The results demonstrated that the clustered ANN models more accurately reproduced the temporal distribution of *Chl-a*. With the 3-cluster ANN models, *R*^*2*^ increased to 0.485, 0.618, 0.634 and 0.545 from 0.345, 0.428, 0.465 and 0.367 at Shinam station, Dasan station, Goryung station and Sangdong station, respectively for 5 years (2013–2017), compared to the non-clustered ANN models.

Using the clustered ANN models, this study assessed the relative importance of the river environmental variables on the seasonal dynamics of *Chl-a*. The results showed that *Q* contributed more than 45% to the predicted *Chl-a* because the water residence time played a vital role in governing the phytoplankton growth mechanism in the flowing water body. In addition, the influence of *Q* tended to amplify with the increase in the water temperature because the artificial mixing induced by the enhanced *Q* strongly impacted the timing and magnitude of cyanobacterial bloom occurring in summer. Whereas, the relative importance of *TP* declined to less than 10% as water temperature increased even if *TP* still worked as the key limiting factors for *Chl-a* belonging to the low water temperature, which represented the diatom abundance, as the relative importance of *TP* increased up to about 30%. Therefore, it can be concluded that the relationship between *Chl-a* and the river environmental factors is temporally heterogeneous in the study reach.

Furthermore, this study proposed the countermeasures against HAB associated with the cyanobacterial bloom, taking into account the effect of the flow control on the HAB reduction with the clustered ANN models. The results of the 3-cluster ANN models illustrated that *Q* of 400 m^3^/s was the optimal level for the HAB mitigation. However, at Goryung station, it was found that the mitigation discharge should increase up to 1.5 times the original level of *Q* owing to the increase in *EC* resulted from the excessive effluents from the Kumho River. Therefore, it was important not only to manage the weir discharge but also to reduce the tributary-induced pollutant sources of *Chl-a* in order to suppress the phytoplankton blooms in the regulated Nakdong River.
